# First report of coexistence of *bla*_KPC-2_-, *bla*_NDM-1_- and *mcr-9*-carrying plasmids in a clinical carbapenem-resistant *Enterobacter hormaechei* isolate

**DOI:** 10.3389/fmicb.2023.1153366

**Published:** 2023-03-23

**Authors:** Qian Yuan, Peiyuan Xia, Lirong Xiong, Linli Xie, Shan Lv, Fengjun Sun, Wei Feng

**Affiliations:** Department of Pharmacy, Southwest Hospital, Third Military Medical University (Army Medical University), Chongqing, China

**Keywords:** *Enterobacter hormaechei*, multidrug resistance, plasmid, carbapenemase genes, *mcr-9*

## Abstract

**Introduction:**

Colistin is regarded as one of the last-resort antibiotics against severe infections caused by carbapenem-resistant Enterobacteriaceae. Strains with cooccurrence of *mcr-9* and carbapenemase genes are of particular concern. This study aimed to investigate the genetic characteristics of a *bla*_KPC-2_-carrying plasmid, *bla*_NDM-1_-carrying plasmid and *mcr-9-*carrying plasmid coexisting in a carbapenem-resistant *Enterobacter hormaechei* isolate.

**Methods:**

*E. hormaechei* strain E1532 was subjected to whole-genome sequencing, and the complete nucleotide sequences of three resistance plasmids identified in the strain were compared with related plasmid sequences. The resistance phenotypes mediated by these plasmids were analyzed by plasmid transfer, carbapenemase activity and antimicrobial susceptibility testing.

**Results:**

Whole-genome sequencing revealed that strain E1532 carries three different resistance plasmids, pE1532-KPC, pE1532-NDM and pE1532-MCR. pE1532-KPC harboring *bla*_KPC-2_ and pE1532-NDM harboring *bla*_NDM-1_ are highly identical to the IncR plasmid pHN84KPC and IncX3 plasmid pNDM-HN380, respectively. The *mcr-9-*carrying plasmid pE1532-MCR possesses a backbone highly similar to that of the IncHI2 plasmids R478 and p505108-MDR, though their accessory modules differ. These three coexisting plasmids carry a large number of resistance genes and contribute to high resistance to almost all antibiotics tested, except for amikacin, trimethoprim/sulfamethoxazole, tigecycline and polymyxin B. Most of the plasmid-mediated resistance genes are located in or flanked by various mobile genetic elements, facilitating horizontal transfer of antibiotic resistance genes.

**Discussion:**

This is the first report of a single *E. hormaechei* isolate with coexistence of three resistance plasmids carrying *mcr-9* and the two most common carbapenemase genes, *bla*_KPC-2_ and *bla*_NDM-1_. The prevalence and genetic features of these coexisting plasmids should be monitored to facilitate the establishment of effective strategies to control their further spread.

## 1. Introduction

Carbapenem-resistant *Enterobacteriaceae* (CRE) bacteria pose a serious threat to global public health owing to rapid emergence of multidrug resistance (MDR) and limited therapeutic agents available (Ma et al., 2023). Production of carbapenemases, especially KPC and NDM, is the main mechanism of carbapenem resistance in CRE clinical isolates (Peng et al., 2022). The *bla*_KPC_ gene is typically located on plasmids of different incompatibility (Inc) groups, such as IncF-, IncI-, IncA/C-, IncX-, and IncR-type plasmids (Chen et al., 2014), and the *bla*_NDM_ gene is mainly carried by IncX3-type plasmids (Zhang et al., 2017; Yoon et al., 2018). These plasmids, which are often easily transferable, can facilitate the spread of *bla*_KPC_ and *bla*_NDM_ resistance genes by horizontal gene transfer among different bacterial populations, complicating clinical therapy and infection control.

Colistin, a cationic polypeptide, is regarded as an antibiotic of last resort for treatment of severe infections caused by CRE (Poirel et al., 2017; Yang et al., 2020). However, the discovery of plasmid-mediated mobile colistin resistance (*mcr*) genes has triggered extensive concern due to the possibility of horizontal transfer of colistin resistance. The *mcr-1* gene was the first *mcr* variant initially reported in China in 2015 in *Escherichia coli* and *Klebsiella pneumoniae* isolates (Liu et al., 2016). To date, various types of *mcr* genes (*mcr-2* to *mcr-10*) have been identified worldwide in Enterobacteriaceae (Ling et al., 2020; Zhang et al., 2022). *mcr-9* is closely related to *mcr-3*, with 65% amino acid identity and 99.5% nucleotide identity (Kieffer et al., 2019). It was first identified in a colistin-susceptible *Salmonella enterica serotype typhimurium* clinical isolate in the United States in 2019 (Carroll et al., 2019) and now has disseminated to various Enterobacteriaceae species, with global distribution in 21 countries across six continents (Li et al., 2020). Surprisingly, reports on coexistence of *mcr-9* and carbapenemase genes (such as *bla*_NDM_, *bla*_VIM_, *bla*_KPC_, *bla*_IMP_ and *bla*_OXA-48_) in Enterobacteriaceae have been increasing worldwide (Chavda et al., 2019; Yuan et al., 2019; Kananizadeh et al., 2020; Liu et al., 2021; Simoni et al., 2021; Yao et al., 2022). These genes may be present in different gene cassettes on a single plasmid or different plasmids from one isolated strain. In this study, we describe cooccurrence of three different MDR plasmids, pE1532-KPC, pE1532-NDM and pE1532-MCR, which carry the *bla*_KPC-2_, *bla*_NDM-1_, and *mcr-9* genes, respectively, in a single carbapenem-resistant *Enterobacter hormaechei* clinical isolate. To the best of our knowledge, this is the first report of a clinical *E. hormaechei* isolate coharboring *mcr-9* and the two most common carbapenemase genes, *bla*_KPC-2_ and *bla*_NDM-1_.

## 2. Materials and methods

### 2.1. Bacterial isolation and identification

*Enterobacter hormaechei* E1532 was isolated from hydrothorax and ascites samples of a patient in a teaching hospital in Henan, China, in 2015. Bacterial species were identified using the VITEK 2 compact system (bioMérieux, France) as well as *16S rRNA* sequencing. The presence of carbapenemase genes (Chen et al., 2015) and *mcr* genes (*mcr-1* to *mcr-10*) (Rebelo et al., 2018; Borowiak et al., 2020; Kim et al., 2021) was screened by PCR amplification using primers described previously, and the positive products were sequenced using an ABI Sequencer (Life Technologies, CA, United States). The genotype of strain E1532 was analyzed using the multilocus sequence typing (MLST) method to amplify and sequence the seven housekeeping genes (*dnaA*, *fusA*, *gyrB*, *leuS*, *pyrG*, *rplB* and *rpoB*) (Miyoshi-Akiyama et al., 2013), and sequence type (ST) was defined on the basis of seven allele numbers available on the MLST website.^1^

### 2.2. Whole-genome sequencing and sequence assembly

Total genomic DNA of *E. hormaechei* E1532 was extracted from cell pellets using a bacterial DNA kit (OMEGA, USA), and the purified DNA was subjected to whole-genome sequencing by a combination of PacBio RS (Pacifc Biosciences, CA, USA) and Illumina NovaSeq (Illumina, CA, USA) sequencing platforms. Paired-end DNA libraries were constructed with an average insert size of 400 bp (ranging from 300 to 500 bp) for Illumina sequencing, and shotgun DNA libraries were generated with a 15 kb insert size (ranging from 10 kb to 20 kb) for PacBio Biosciences sequencing. Clean reads were obtained after filtering the low-quality sequence data and then *de novo* assembled by Unicycler software (Wick et al., 2017). The Illumina-generated short reads were utilized to correct the PacBio-generated long reads using the Pilon tool (Walker et al., 2014).

### 2.3. Genome annotation and bioinformatics analysis

Prediction and annotation of coding genes and pseudogenes was carried out using the RAST 2.0 algorithm (Brettin et al., 2015). Putative open reading frames (ORFs) were further assessed for functions by BLASTP and BLASTN (Boratyn et al., 2013) against the NCBI RefSeq (O’Leary et al., 2016) and UniProtKB/Swiss-Prot (Boutet et al., 2016) databases. The precise species assignment was further confirmed based on whole-genome sequencing using average nucleotide identity (ANI) analysis with the JSpeciesWS server. PlasmidFinder (Carattoli et al., 2014) was used to examine plasmid replicon type. The presence of antibiotic resistance genes, insertion sequences (ISs), transposons and integrons was analyzed *in silico* using the ResFinder (Bortolaia et al., 2020), CARD (Jia et al., 2017), ISfinder (Siguier et al., 2006), Tn Number Registry (Roberts et al., 2008) and INTEGRALL (Moura et al., 2009) databases. BLASTN and MUSCLE 3.8.31 (Edgar, 2004) were employed for alignment and comparison of the plasmid sequences analyzed in this study with highly homologous plasmid sequences publicly available in NCBI. The circular graph of plasmid sequences and linear comparative graph were constructed by Inkscape 0.48.1 software.^2^

### 2.4. Plasmid transfer

The filter mating method was used for the plasmid conjugation assay (Ouyang et al., 2018). Briefly, rifampicin-resistant *E. coli* EC600 was used as the recipient. Equal amounts of donor and recipient strains were mixed together, and mating was performed on the filter membranes of brain heart infusion (BHI) agar plates for 12–18 h at 37°C. The mixtures were spread on doubly selective agar plates containing rifampin together with indicated additional antibiotics for selecting an *E. coli* transconjugant carrying one of the following resistance markers: imipenem for *bla*_KPC_ (pE1532-KPC) and *bla*_NDM_ (pE1532-NDM), and azithromycin for *mph*(A) (pE1532-MCR). The presence of the resistance markers carried by transconjugants was confirmed by PCR amplification and sequencing.

For the electrotransformation experiment, streptomycin- and tetracycline-resistant *E. coli* TOP10 was selected as the recipient. Plasmid DNA was extracted using QIAGEN Plasmid Midi Kit (Qiagen, Germany) and transformed into TOP10 competent cells by electroporation. After reviving the bacterial cells for 1 h at 37°C and 200 rpm, positive electroporants carrying the *bla*_KPC_ or *bla*_NDM_ or *mph*(A) gene were selected on super optimal broth (SOB) agar plates containing imipenem or azithromycin, and further verified by PCR and sequence analysis.

### 2.5. Carbapenemase activity assay

The class A/B/D carbapenemase activity of the E1532 strain as well as its transconjugant and electroporant was assessed by the CarbaNP test as described in our previous study (Feng et al., 2022). Overnight bacterial cultures were seeded into MH broth supplemented with 4 μg/ml imipenem and incubated with continuous shaking until the bacterial density reached 1.0–1.4. Cell pellets were collected by centrifugation and subjected to washing with Tris–HCl twice. The sonication process was performed to lyse the bacterial cells. The supernatant from the cell lysis solution was added to substrates I–V at a ratio of 1:1 and then allowed to interact at 37°C for 1–2 h. The phenotypic results of carbapenemase activity were observed by color changes of the mixture.

### 2.6. Antimicrobial susceptibility testing

Minimum inhibitory concentrations (MICs) were determined using VITEK 2 System and AST-N334 cards for the following antimicrobial agents: amoxicillin/clavulanic acid, piperacillin/tazobactam, cefuroxime, cefuroxime axetil, cefoxitin, ceftazidime, ceftriaxone, cefoperazone/sulbactam, cefepime, ertapenem, imipenem, amikacin, levofloxacin, tigecycline and trimethoprim/sulfamethoxazole. For polymyxin B, the susceptibility test was performed with the broth microdilution method. The resistance results for tigecycline and colistin were judged according to the breakpoints of European Committee on Antimicrobial Susceptibility Testing (EUCAST)^3^; the breakpoints of other antibiotics were defined following the standard of the Clinical and Laboratory Standards Institute (CLSI). The *E. coli* standard strain ATCC 25922 served as the quality control for susceptibility testing.

### 2.7. Nucleotide sequence accession numbers

The complete sequences of chromosomes and plasmids have been submitted to the GenBank database under accession numbers CP114571 (chromosome), CP114573 (pE1532-KPC), CP114574 (pE1532-MCR), CP114575 (pE1532-NDM), and CP114572 (pE1532-4).

## 3. Results and discussion

### 3.1. *Enterobacter hormaechei* E1532 coharboring *bla*_KPC-2_, *bla*_NDM-1_ and *mcr-9* genes

*E. hormaechei* E1532 was found to be resistant to all penicillins, cephalosporins, carbapenems, and fluoroquinolones and showed intermediate resistance to tigecycline; it remained sensitive to amikacin, polymyxin B and trimethoprim/sulfamethoxazole (Table 1). PCR screening and sequencing identification showed that *E. hormaechei* E1532 harbors two carbapenemase genes, *bla*_KPC-2_ and *bla*_NDM-1_, and a newly identified colistin resistance gene, *mcr-9*. MLST analysis revealed that strain E1532 belongs to ST93 (allelic profile: 9–4–14-61-37-4-9), which is a globally disseminated high-risk clone that is frequently reported in China (Peirano et al., 2018; Zhao et al., 2020; Chen et al., 2021).

**Table 1:** Antimicrobial susceptibility profiles of *Enterobacter hormaechei* E1532 and its transconjugants and electroporants.

Category	Antibiotic	MIC(μg/ml)/antimicrobial susceptibility					
		E1532	pE1532-KPC-TOP10	pE1532-NDM-EC600	pE1532-NDM-TOP10	TOP10	EC600
Penicillins	Amoxicillin/clavulanic acid	≥32/R	≥32/R	≥32/R	≥32/R	4/S	≤4/S
	Piperacillin/tazobactam	≥128/R	≥128/R	≥128/R	≥128/R	≤4/S	≤4/S
Cephalosporins	Cefuroxime	≥64/R	≥64/R	≥64/R	≥64/R	8/S	16I
	Cefuroxime axetil	≥64/R	≥64/R	≥64/R	≥64/R	4/S	16I
	Cefoxitin	≥64/R	≥64/R	≥64/R	≥64/R	≤4/S	≤4/S
	Ceftazidime	≥64/R	32/R	≥64/R	≥64/R	≤1/S	≤1/S
	Ceftriaxone	≥64/R	≥64/R	≥64/R	≥64/R	≤1/S	≤1/S
	Cefoperazone/sulbactam	≥64/R	≥64/R	≥64/R	≥64/R	≤1/S	≤1/S
	Cefepime	≥32/R	16/R	16/R	16/R	≤1/S	≤1/S
Carbapenems	Ertapenem	≥8/R	≥8/R	≥8/R	≥8/R	≤1/S	≤1/S
	Imipenem	≥16/R	8/R	≥16/R	8/R	≤1/S	≤1/S
Fluoroquinolones	Levofloxacin	≥8/R	≤0.25/S	0.5/S	≤0.25/S	≤0.25/S	0.5/S
Aminoglycosides	Amikacin	≤2/S	≤2/S	≤2/S	≤2/S	≤2/S	≤2/S
Glycylcycline	Tigecycline	4/I	≤0.5/S	≤0.5/S	≤0.5/S	≤0.5/S	≤0.5/S
Sulfanilamides	Trimethoprim/sulfamethoxazole	40/S	≤20/S	≤20/S	≤20/S	≤20/S	≤20/S
Lipopeptide	Polymyxin B	0.25/S	0.25/S	0.25/S	0.25/S	0.25/S	0.25/S

MIC = minimum inhibitory concentration; S = sensitive; R = resistant; I = intermediate.

Whole-genome sequencing revealed that strain E1532 has a single circular chromosome sequence of 4,869,794 bp with an average G + C content of 55.31% and contains a total of 4,717 predicted ORFs. A total of four plasmids, namely, pE1532-KPC, pE1532-NDM, pE1532-MCR and pE1532-4, were present in strain E1532, with circular closed DNA sequences of 39,461 bp, 53,769 bp, 308,217 bp, and 69,180 bp in length with 44, 62, 360, and 90 predicted ORFs, respectively (Supplementary Figure S1; Table 2). Each plasmid consists of backbone regions (responsible for plasmid maintenance, replication and/or conjugal transfer) and one or more accessory modules (acquired DNA regions associated with and bordered by mobile elements) inserted at different sites of the backbone regions (Supplementary Figure S1; Table 2). Antibiotic resistance genes were identified using ResFinder and CARD analysis. The chromosome of E1532 carries three intrinsic resistance genes involved in resistance to β-lactams (*bla*_ACT-7_), aminoglycoside [*aph(3′)-Ia*] and fosfomycin (*fosA*). Plasmids pE1532-KPC, pE1532-NDM and pE1532-MCR harbor a total of 19 genes conferring resistance to β-lactams (*bla*_KPC-2_, *bla*_NDM-1_, *bla*_SHV-12_, *bla*_DHA-1_, *bla*_TEM-1_), aminoglycosides [*aacC2, aphA1, aph(6)-Id, aph(3″)-Ib*], tetracyclines [*tet*(A)], bleomycin (*bla*_MBL_), macrolide [*mph*(A)], fluoroquinolones (*qnrB4*), sulfonamide (*sul1*), tunicamycin (*tmrB*), colistin (*mcr-9*), quaternary ammonium (*qacED1*), tellurium (the *ter* locus), and mercuric (the *mer* locus) (Table 3). However, no resistance gene was found on plasmid pE1532-4, which only harbors a Tn*3*-family transposon remnant.

**Table 2:** Genomic features of the four plasmids carried by strain E1532.

Feature	pE1532-KPC	pE1532-NDM	pE1532-MCR	pE1532-4
Incompatibility group	IncR	IncX3	IncHI2	IncFII
Total length (bp)	39,461	53,769	308,217	69,180
Total number of ORFs	44	62	360	90
Mean G + C content, %	55.67	49.08	47.48	52.97
Length of the backbone (bp)	12,034	34,724	230,863	69,180
Accessory modules	The *bla*_KPC-2_ region	The *bla*_NDM-1_ region and IS*Kox3*	The MDR-1 region, the MDR-2 region, IS*Cfr9*-IS*Cfr15*, IS*1B*-IS*Kpn26*, the IS*Kpn2*1:∆Tn*6363* region, three separate copies of IS*903B*, IS*Kpn26*, IS*5*, Tn*6362*, Tn*2* and ∆IS*903B*	The Tn*3*-family transposon remnant

**Table 3:** Drug resistance genes in plasmids analyzed.

Plasmid	Resistance gene	Resistance phenotype	Nucleotide position	Accessory or backbone region located
pE1532-KPC	*bla*_KPC-2_	β-lactam resistance	25,914-26,795	The *bla*_KPC-2_ region
	*tet*(A)	Tetracycline resistance	15,880-17,079	
pE1532-NDM	*bla*_NDM-1_	β-lactam resistance	17,826-18,638	The *bla*_NDM-1_ region
	*bla*_SHV-12_	β-lactam resistance	9,324-10,183	
	*bla*_MBL_	Bleomycin resistance	17,457-17,822	
pE1532-MCR	The *ter* locus	Tellurium resistance	64,703-84,651	Backbone region
	*aacC2*	Aminoglycoside resistance	156,517-157,377	The MDR-1 region
	*bla*_DHA-1_	β-lactam resistance	137,136-138,275	
	*bla*_SHV-12_	β-lactam resistance	145,679-146,539	
	*bla*_TEM-1_	β-lactam resistance	150,685-151,545	
	*mph*(A)	Macrolide resistance	158,436-159,341	
	*qacED1*	Quaternary ammonium resistance	139,376-139,858	
	*qnrB4*	Fluoroquinolone resistance	132,368-133,015	
	*sul1*	Sulfonamide resistance	139,852-140,691	
	*tmrB*	Tunicamycin resistance	155,962-156,504	
	*strA*	Aminoglycoside resistance	263,392-264,228	The MDR-2 region
	*strB*	Aminoglycoside resistance	264,228-265,031	
	*mcr-9*	Colistin resistance	252,943-254,562	
	*aphA1*	Aminoglycoside resistance	224,978-225,793	The IS*Kpn21*:∆Tn*6363* region
	*bla*_TEM-1_	β-lactam resistance	115,557-116,417	Tn*2*
	The *mer* locus	Mercuric resistance	105,015-108,991	Tn*6362*

Plasmid pE1532-NDM was successfully transferred to *E. coli* by electrotransformation and conjugation experiments, obtaining the corresponding electroporant pE1532-NDM-TOP10 and transconjugant pE1532-NDM-EC600 (Table 1). In order to investigate the transmissible possibility of conjugative plasmid pE1532-NDM in different hosts, the conserved backbone sequences of pE1532-NDM were aligned by BLASTN with the *bla*_NDM-1_-harboring IncX3 plasmids available in GenBank. Among the top 100 plasmids with the highest backbone sequence similarity of pE1532-NDM, the plasmids were selected based on the origin of bacterial species or hosts different from pE1532-NDM. A total of 11 plasmids were included and they were mainly isolated from bacterial strains from human. In addition, there were also four plasmids from shrimp, chicken, hospital sewage and unknown source, respectively (Supplementary Table S1). The results of our analysis showed that IncX3 plasmids have an extensive host range. However, only the electroporant pE1532-KPC-TOP10, and not the transconjugant, was obtained by transferring pE1532-KPC into *E. coli*, which may be because pE1532-KPC lacks the conjugal transfer genes in backbone regions (Supplementary Figure S1) and is not self-transmissible (Compain et al., 2014; Jing et al., 2019). Repeated attempts failed to transfer pE1532-MCR into *E. coli* through conjugation and electroporation; this may be attributed to the following facts: (1) pE1532-MCR is a 308 kb megaplasmid which limits the success of conjugation and electroporation. (2) the insertion event occurred within the two conjugal transfer regions *tra1* and *tra2* might render this plasmid nonconjugative (Supplementary Figure S1). In a carbapenemase activity assay, strain E1532 showed class A + B activity, the pE1532-NDM-harboring electroporant and transconjugant class B activity, and the pE1532-KPC-harboring electroporant A activity (data not shown). Phenotypic susceptibility testing showed these electroporants and transconjugant to be resistant to all β-lactams tested, including carbapenems (Table 1), which was consistent with the presence of carbapenemase genes in these strains.

### 3.2. Comparative genomics of *bla*_KPC-2_-carrying plasmid pE1532-KPC

**Figure 1:**
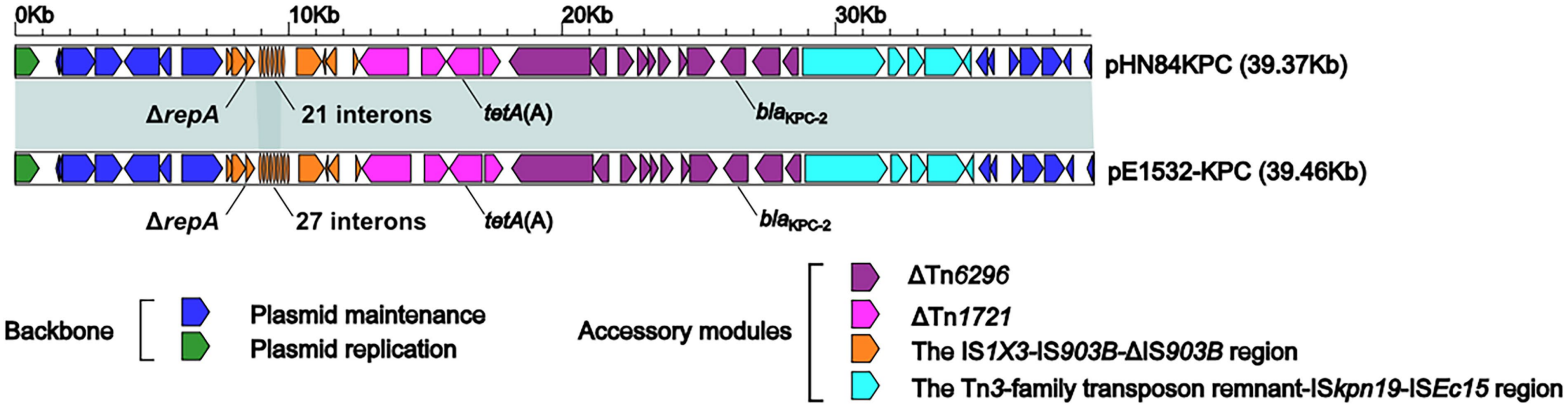
Linear comparison of plasmid pE1532-KPC with pHN84KPC (accession number KY296104). Genes are denoted by arrows. Genes, mobile elements and other features are colored based on function classification. Shading denotes shared regions of homology (>95% nucleotide identity).

The entire sequence of pE1532-KPC is highly similar to that of the reference IncR plasmid pHN84KPC (accession number KY296104), with 100% query coverage and > 99% nucleotide identity (Figure 1). pE1532-KPC and pHN84KPC share the most complete IncR backbone gene loci encoding plasmid replication initiation (*repB*) and plasmid maintenance (*parAB*, *umuCD*, *retA, vagCD*, *resD*). A single accessory module, the *bla*_KPC-2_ region containing two resistance genes, *bla*_KPC-2_ and *tet*(A), is inserted at a site between *retA* and *vagD* in the backbone of these two plasmids (Supplementary Figure S1). The *bla*_KPC-2_ region contains the *tet*(A)-carrying ΔTn*1721* cassette (Allmeier et al., 1992) and the *bla*_KPC-2_-carrying ΔTn*6296* cassette (Jiang et al., 2010) flanked by the upstream IS*1X3*-IS*903B*-ΔIS*903B* region and the downstream Tn*3*-family transposon remnant-IS*Kpn19*-ΔIS*Ec15* region (Figure 1). The only modular difference between these two plasmids is with regard to the copy number (21 in pHN84KPC, 27 in pE1532-KPC) of the 37-bp tandem repeat within interons, which is located behind the truncated replication initiation gene *ΔrepA* in the *bla*_KPC-2_ region.

### 3.3. Comparative genomics of *bla*_NDM-1_-carrying plasmid pE1532-NDM

**Figure 2:**
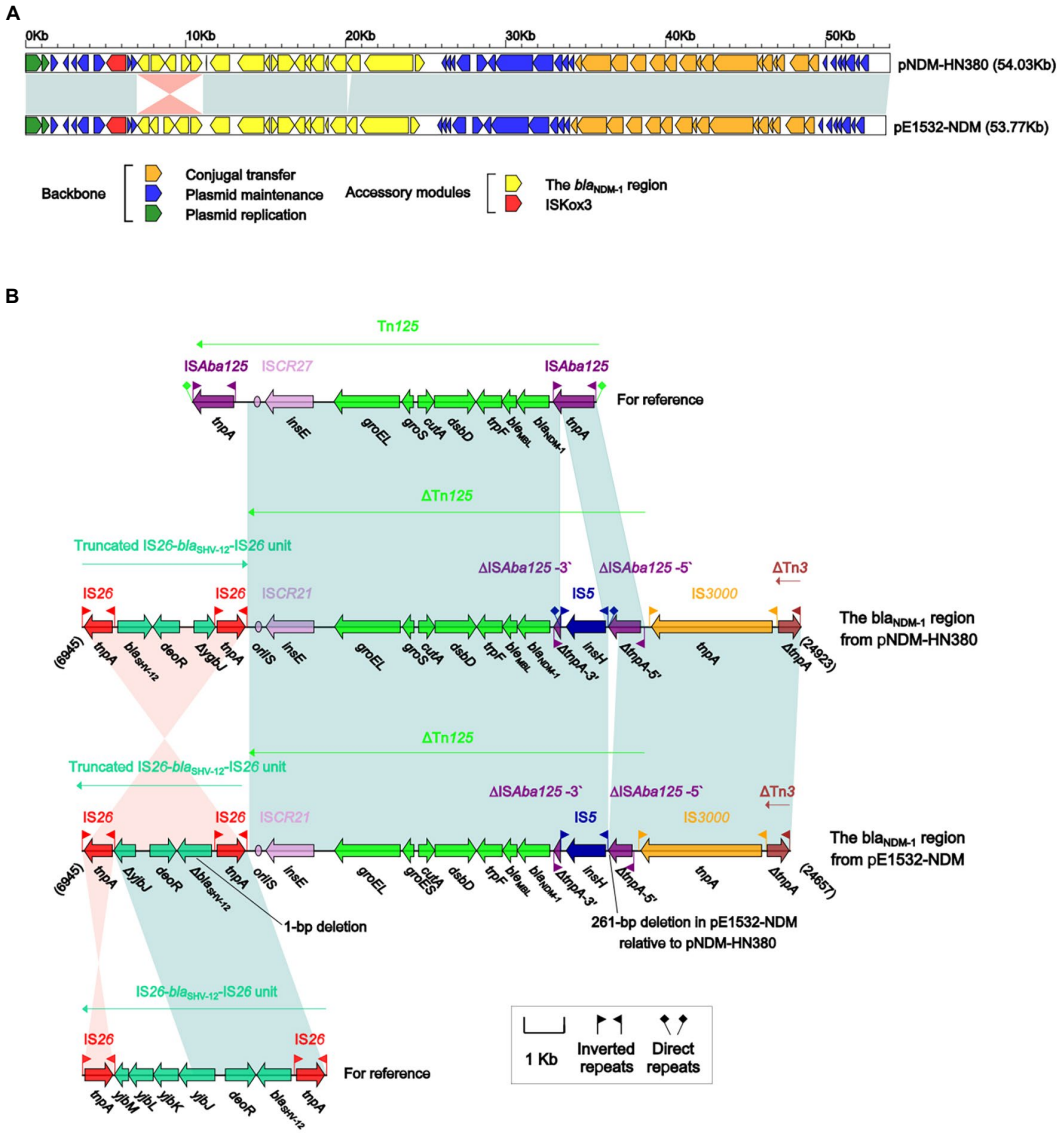
Comparison of plasmid pE1532-NDM with pNDM-HN380. **(A)** Linear comparison of the two sequenced plasmids pE1532-NDM and pNDM-HN380 (accession number JX104760). **(B)** The *bla*_NDM-1_ region from pE1532-NDM and comparison with related regions. Genes are denoted by arrows. Genes, mobile elements and other features are colored based on functional classification. Shading denotes shared regions of homology (≥90% nucleotide identity). Numbers in brackets indicate nucleotide positions within the corresponding plasmids. The accession numbers of the Tn*125* and IS*26-bla*_SHV-12_-IS*26* unit for reference are JN872328 and CP003684, respectively.

pE1532-NDM displays >99% nucleotide identity (with 100% query coverage) with the reference IncX3 plasmid pNDM-HN380 (Ho et al., 2012) obtained from *K. pneumoniae* isolate CRE380 in China (Figure 2A). pE1532-NDM and pNDM-HN380 have identical backbones that share a set of core genes for plasmid replication (*repB* and *bis*), partition (*parA*), maintenance (*topB* and *stpA*) and conjugal transfer (*pilX* genes) (Supplementary Figure S1). Each of them harbors two accessory modules, IS*Kox3* and the *bla*_NDM-1_ region, which are inserted at different sites of the plasmid backbone. All resistance genes are located in the *bla*_NDM-1_ region. This accessory resistance region (18 kb in length) originated sequentially as a truncated IS*26-bla*_SHV-12_-IS*26* unit, *bla*_NDM-1_-carrying ΔTn*125*, IS*3000* and ΔTn*3* (Figure 2B). The truncated IS*26-bla*_SHV-12_-IS*26* unit was generated by deletion of *yjbJ* (partial)-*yjbK*-*yjbL*-*yjbM* genes and inversion of IS*26* at the 3′ region from the prototype composite transposon-like IS*26-bla*_SHV-12_-IS*26* unit (Ford and Avison, 2004). ΔTn*125* is a derivative of IS*Aba125*-flanked composite transposon Tn*125* (Poirel et al., 2012), lacking the IS*Aba125* element at the 3′ region and having an interrupted and truncated IS*Aba125* at the 5′ region by insertion of an IS*5* element.

At least four major module differences were identified between the *bla*_NDM-1_ regions of pE1532-NDM and pNDM-HN380 (Figure 2B). First, the truncated IS*26-bla*_SHV-12_-IS*26* unit in pE1532-NDM is inverted compared to pNDM-HN380. Second, the *bla*_SHV-12_ gene cannot form an ORF due to the absence of 1 bp, making it a pseudogene. Third, a 261-bp deletion at the 5′-terminal region of ΔIS*Aba125* was found in pE1532-NDM. Fourth, direct repeats (DRs) have been lost at both ends of the IS*5* element in pE1532-NDM.

### 3.4. Comparative genomics of the *mcr-9*-carrying plasmid pE1532-MCR

The pE1532-MCR backbone is closely related to the first prototype IncHI2 plasmid R478 (Gilmour et al., 2004) from *Serratia marcescens*, with 95% query coverage and > 99% nucleotide identity, and to another IncHI2 plasmid, p505108-MDR (Shi et al., 2018) from *Cronobacter sakazakii*, with 98% query coverage and > 99% nucleotide identity (Supplementary Figure S2). These three plasmids share the core IncHI2 backbone, including the regions responsible for plasmid replication (*repHIA*, *repHI2*), partition (*parAB*, *parMR*) and conjugal transfer (*tra1* and *tra2* regions) (Supplementary Figure S1).

Linear comparison revealed that the following different regions are present among the backbones of pE1532-MCR, R478 and p505108-MDR (Supplementary Figure S2A). (1) An IS*903B* element in pE1532-MCR but a ΔIS*903D* element in p505108-MDR is inserted between *parR* and *htdA* relative to R478, leading to the interruption of the conjugal transfer region *tra2*. (2) An IS*Kpn26* element is inserted between the backbone genes *orf2034* and *orf222* in pE1532-MCR. (3) An IS*5* element is inserted between *orf1161* and *hha* in pE1532-MCR. (4) Two backbone regions, *hipB*-*hipA*-*orf207*-*orf411* and *orf189*-*orf426*-*orf258*, are inserted between *orf1389* and *orf609* in pE1532-MCR and p505108-MDR. (5) *orf159* is interrupted by a Tn*2* element in pE1532-MCR and p505108-MDR. (6) The *klaB* to *orf534* region is truncated by insertion of the MDR-2 region in pE1532-MCR and p505108-MDR relative to R478, leaving only the remnant of the *klaB* and *orf534* genes. (7) An IS*903B* element in pE1532-MCR but a ΔIS*903D* element in p505108-MDR is inserted between *orf2385* and *orf450*, leading to the truncation of both genes. (8) A 377-bp insertion at the 3′-end of *orf444* resulted in the replacement of *orf444* with *orf588* in pE1532-MCR and p505108-MDR. (9) The MDR-1 and MDR-2 regions are inserted into the boundary of *ΔklaB* and *orf819*, respectively, with inversion of the entire backbone region between *ΔklaB* and *orf819* in pE1532-MCR.

To more clearly and intuitively observe the genetic features of the backbone region between *ΔklaB* and *orf819* in these three plasmids, the *ΔklaB* to *orf819* region in pE1532-MCR was reverted compared with that of R478 and p505108-MDR (Supplementary Figure S2B). The genetic differences among them were as follows: (1) An IS*150* element is located between the two backbone genes *orf198* and *ldrB* in R478 but replaced by both the IS*Cfr9*-IS*Cfr15* region and the *orf612*-*fieF*-*relB*-*relE* backbone region in pE1532-MCR and p505108-MDR. (2) IS*186B* is lost between *ldrB* and *orf321* in pE1532-MCR and p505108-MDR relative to R478. (3) Tn*10* is inserted into *orf300* in R478. (4) The *traI* gene in the conjugal transfer region *tra1* is interrupted by a truncated IS*903B* in pE1532-MCR. (5) The IS*1B*-IS*Kpn26* region and IS*Kpn21-*∆Tn*6363* region are inserted into the *mucA* to *orf1404* backbone region in pE1532-MCR, resulting in deletion of backbone genes between *orf318-1* and *mucA* as well as truncation of *mucA* relative to R478; acquisition of the *aphA1a* region in p505108-MDR causes loss of the *orf318-2* to *retA* backbone region as well as truncation of *mucA*. (6) *orf258* is interrupted by an IS*903B* element in pE1532-MCR.

**Figure 3:**
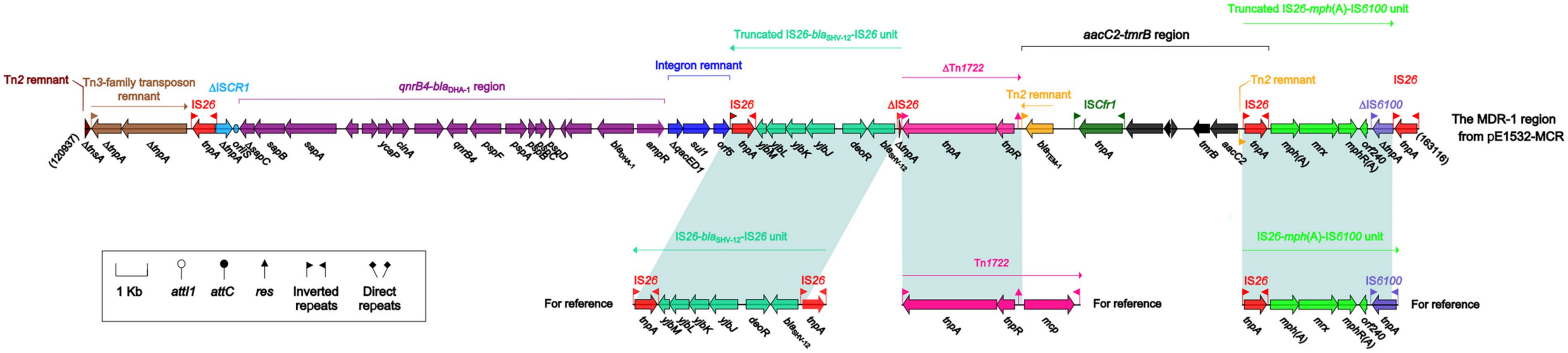
The MDR-1 region from pE1532-MCR and comparison with related regions. Genes are denoted by arrows. Genes, mobile elements and other features are colored based on functional classification. Shading denotes shared regions of homology (≥90% nucleotide identity). Numbers in brackets indicate nucleotide positions within the corresponding plasmids. The accession numbers of the IS*26-bla*_SHV-12_-IS*26* unit, IS*26*-*mph*(A)-IS*6100* unit and Tn*1722* for reference are CP003684, KY270852 and X61367, respectively.

**Figure 4:**
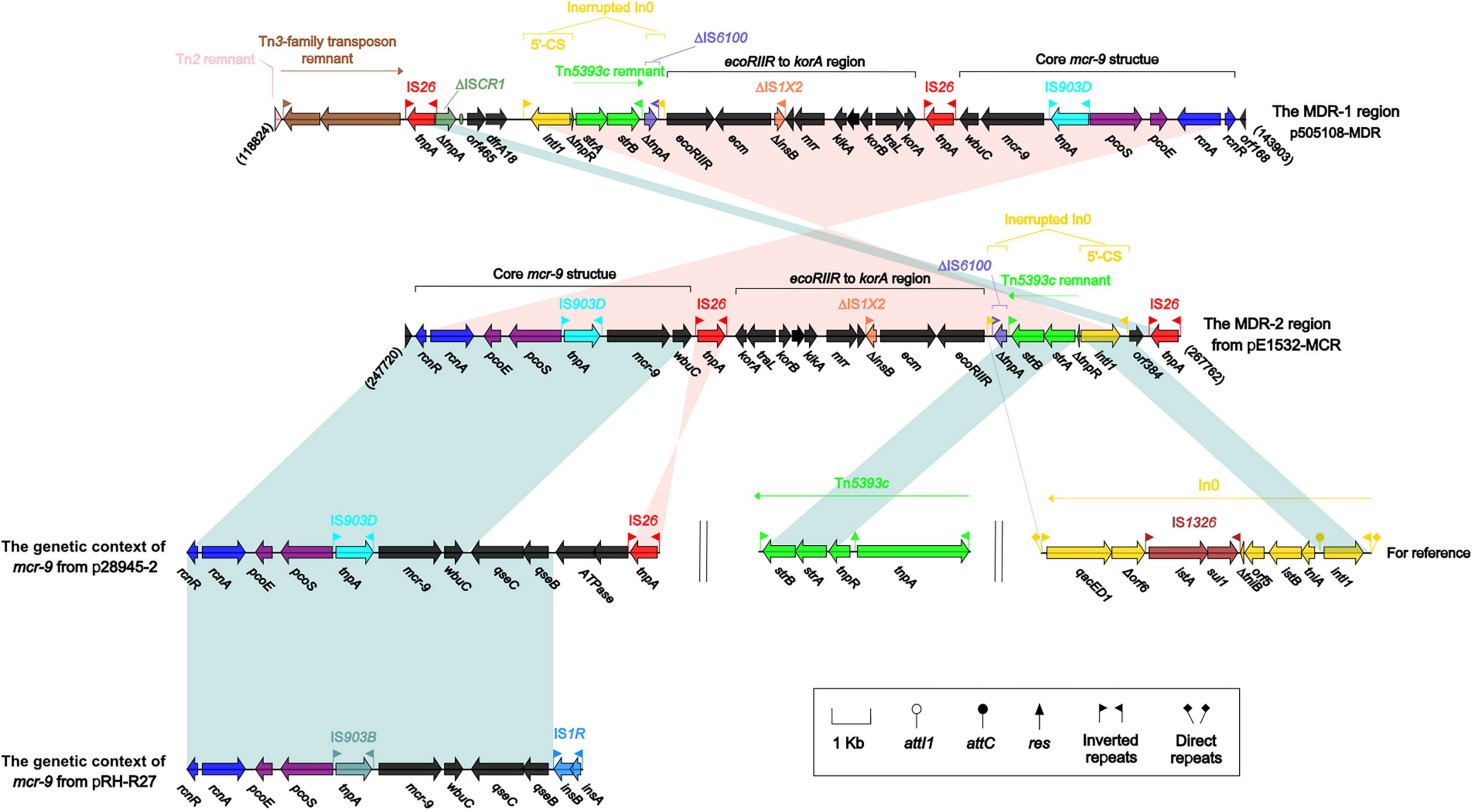
The MDR-2 region from pE1532-MCR and comparison with related regions. Genes are denoted by arrows. Genes, mobile elements and other features are colored based on functional classification. Shading denotes shared regions of homology (≥90% nucleotide identity). Numbers in brackets indicate nucleotide positions within the corresponding plasmids. The accession numbers of Tn*5393c* and In0 for reference are AF262622 and U49101, respectively.

**Figure 5:**
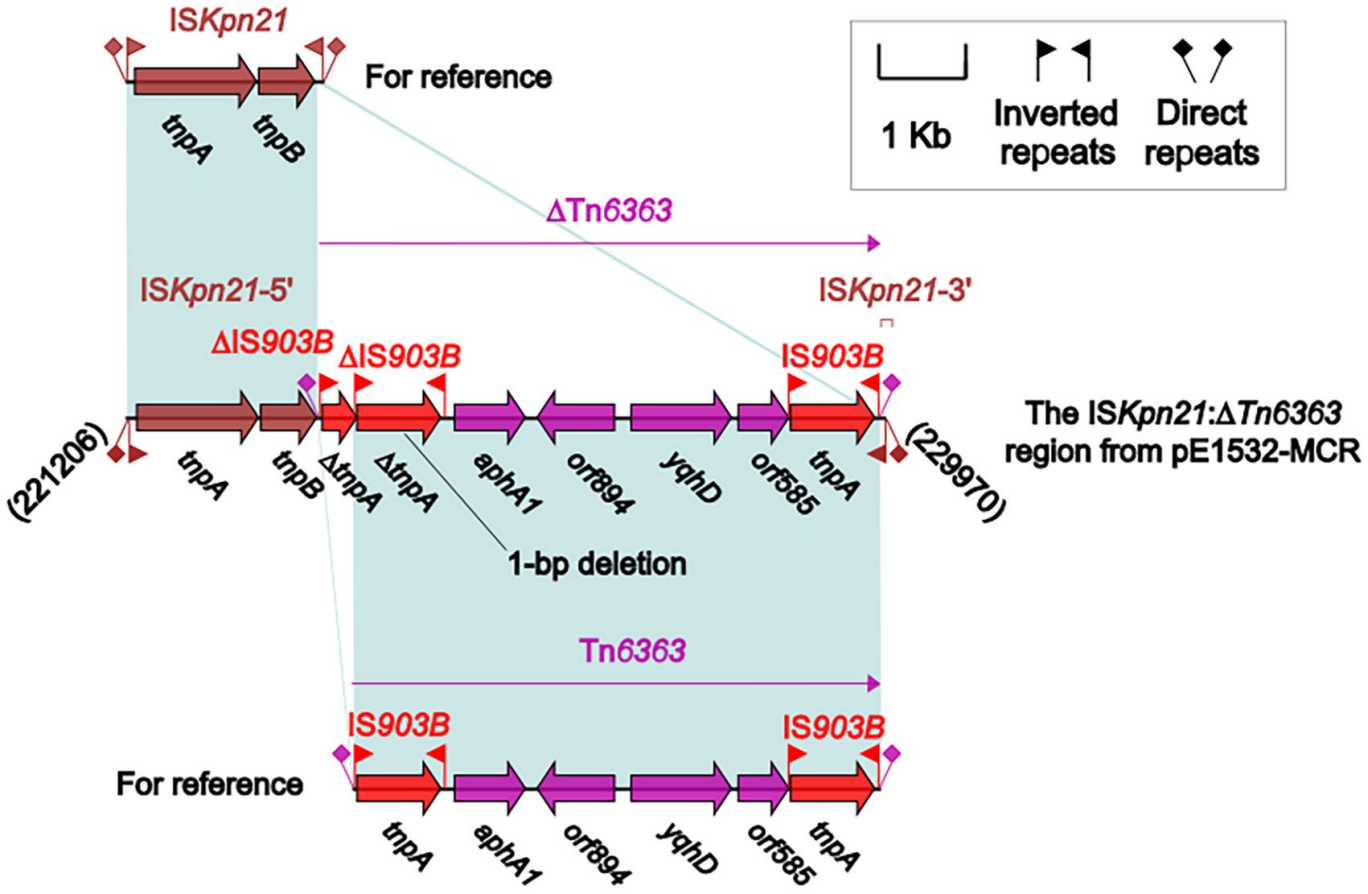
The IS*Kpn2*1:∆Tn*6363* region from pE1532-MCR and comparison with related regions. Genes are denoted by arrows. Genes, mobile elements and other features are colored based on functional classification. Shading denotes shared regions of homology (≥90% nucleotide identity). Numbers in brackets indicate nucleotide positions within the corresponding plasmids. The accession numbers of IS*Kpn2*1 and Tn*6363* for reference are AP012055 and KY978630, respectively.

The differences described above involve not only backbone regions but also accessory modules. pE1532-MCR carries a total of 13 accessory modules, and resistance genes are located in the MDR-1 region (Figure 3), the MDR-2 region (Figure 4), the IS*Kpn2*1:∆Tn*6363* region (Figure 5), Tn*6362* (Supplementary Figure S3) and Tn*2* (Supplementary Figure S3).

### 3.5. The MDR-1 region of pE1532-MCR

The MDR-1 region (Figure 3) harbors five antibiotic resistance loci: *qnrB4*-*bla*_DHA-1_ region (Yim et al., 2013), integron remnant carrying *ΔqacED1* and *sul1*, a truncated IS*26*-*bla*_SHV-12_-IS*26* unit (Ford and Avison, 2004), *aacC2*-*tmrB* region (Partridge et al., 2012), and a truncated IS*26*-*mph*(A)-IS*6100* unit (Liang et al., 2017). IS*26*-*bla*_SHV-12_-IS*26* is an IS*26*-flanked extended-spectrum β-lactamase (ESBL) resistance unit lacking direct repeats (DRs) at both ends. A number of IS*26*-based resistance units have been reported with diverse resistance genes (Partridge, 2011). IS*26* can mediate movement of antibiotic resistance genes and formation of composite transposons, which contribute to MDR region assembly (Harmer and Hall, 2016; Harmer et al., 2020). The IS*26*-*bla*_SHV-12_-IS*26* unit in the MDR-1 region has undergone truncation of the upstream IS*26* element and deletion of its 14-bp IRR (inverted repeat right).

Tn*1722* belongs to a Tn*3*-family unit transposon harbored in the tetracycline resistance transposon Tn*1721* (Allmeier et al., 1992) and includes *tnpA*, *tnpR*, *res* and *mcp* genes bounded by 38-bp IRL (inverted repeat left) and IRR. The ΔTn*1722* element in the MDR-1 region is a derivative of Tn*1722* with deletion of *mcp* and IRR, which is also found in pCRE3-KPC (Dong et al., 2020) and plasmid unnamed3 (accession number CP027150). The IS*26*-*mph*(A)-IS*6100* unit contains a macrolide resistance region *mph*(A)-*mrx*-*mphR*(A) (Noguchi et al., 2000) bracketed by IS*26* and IS*6100* elements. These two IS elements, which have almost identical 14-bp IRs, belong to the IS*6* family, and the homologous recombination mediated by them promotes integration of this resistance unit into the MDR region, similar to the IS*26*-bound resistance unit.

### 3.6. The MDR-2 region of pE1532-MCR

The MDR-2 region of pE1532-MCR (Figure 4) is highly similar to the MDR-1 region of p505108-MDR (97% query coverage and > 99% nucleotide identity), with a reverse array of the *orf168* to *intI1* region and with the same orientated IS*26* downstream of interrupted In0. Both MDR regions harbor a *mcr-9* gene, and the genetic environments of *mcr-9* are identical to each other. *Mcr-9* is flanked by IS*903D* and IS*26* elements, both belonging to the IS*6* family, which play a vital role in dissemination of resistance genes. The genes upstream of *mcr-9* include *rcnR, rcnA, pcoE* and *pcoS*. However, only the *wbuC* gene is located downstream of *mcr-9*, and a two-component system encoding the genes *qseB* and *qseC*, which are involved in *mcr-9* expression (Kieffer et al., 2019), was not found. The lack of *qseB*-*qseC* regulatory genes in plasmid pE1532-MCR may explain the phenomenon that strain E1532 carrying *mcr-9* is sensitive to polymyxin B. The gene composition arrayed as *rcnR-rcnA-pcoE-pcoS-*IS*903-mcr-9-wbuC* is the core structure of *mcr-9* resistance cassettes in the *mcr-9*-carrying IncHI2 plasmids from different Enterobacteriaceae (Li et al., 2020). The different encoding genes and IS elements located downstream of *mcr-9* are the main causes leading to the diverse genetic context of *mcr-9* in IncHI2 plasmids.

In addition to *mcr-9*, other resistance genes (*strA*, *strB*) are present in the MDR-2 region. The class 1 integron In0 (accession number U49101) from *Pseudomonas aeruginosa* plasmid pVS1 is an ancestor of more complex integrons with a weak PcW promoter and an unoccupied integration site *attI* in 5′-CS but no gene cassette array (Bissonnette and Roy, 1992). ΔIn0 from the MDR region of pE1532-MCR/p505108-MDR contains the 5′-CS composed of the integrase gene *intI1* and promoter gene and is bordered by IRi (inverted repeat at the integrase end) and IRt (inverted repeat at the *tni* end), which resulted from insertion of ΔIS*6100* and Tn*5393c* remnant (Tn*3*-family transposon) carrying the aminoglycoside resistance genes *strA and strB* (L’Abee-Lund and Sorum, 2000).

### 3.7. The IS*Kpn21*:ΔTn*6363* region of pE1532-MCR

The IS*Kpn21*:ΔTn*6363* region (Figure 5) is composed of IS*Kpn21* and ΔTn*6363* and has undergone two different transposition events: (1) The ISNCY family element IS*Kpn21* with an IRL-*tnpA*-*tnpB*-IRR structure is integrated at a site between the two backbone genes *orf318-2* and *orf1404* in pE1532-MCR and bordered by 5-bp DRs. (2) The transposon ΔTn*6363* is inserted at a site between *tnpB* and the IRR of IS*Kpn21*, splitting IS*Kpn21* into two parts and leaving 9-bp DRs at both ends of ΔTn*6363*. Tn*6363* (accession number KY978628) is an IS*903B*-flanked composite transposon that possesses an IS*903B*-*aphA1*-*orf894*-*yqhD*-*orf585*-IS*903B* structure (Shi et al., 2018). Insertion of an additional element ΔIS*903B* resulted in a 1-bp deletion of IS*903B* upstream of Tn*6363*, thus generating ΔTn*6363* with three IS*903B* elements. ΔTn*6363* is a derivative of Tn*6363* which carries aminoglycoside resistance gene *aphA1*, the embedding of ΔTn*6363* into IS*Kpn21* mediated by IS*903B* facilitates the horizontal transfer and transmission of antibiotic resistance gene in bacterial populations.

## 4. Conclusion

This is the first report of coexistence of the *bla*_KPC-2_-carrying IncR plasmid, *bla*_NDM-1_-carrying IncX3 plasmid and *mcr-9*-carrying IncHI2 plasmid recovered from the ST93 multidrug-resistant *E. hormaechei* clinical isolate E1532. These three coexisting MDR plasmids carry a large number of resistance genes, rendering the E1532 isolate resistant to almost all antibiotics tested, including carbapenems. The *mcr-9* gene, which is involved in resistance to the last-resort antibiotic, should be given sufficient attention because it has become widely disseminated worldwide among various species of Enterobacteriaceae. Therefore, epidemiological analysis should be performed to monitor the spread of *mcr-9*-positive strains. Moreover, most of these plasmid-mediated resistance genes are located in or flanked by various mobile genetic elements, such as transposons, insertion sequences and integrons, which facilitate acquisition and horizontal transfer of antibiotic resistance genes across bacterial populations. Not all plasmids can transfer themselves, but non-conjugative plasmids can be mobilized with the help of other conjugative plasmids present in the same donor cell. Therefore, there is the possibility of cotransfer of *bla*_KPC-2_-, *bla*_NDM-1_-, and *mcr-9*-carrying plasmids. The main limitation in this study is that we did not apply the conjugation assay to evaluate the cotransfer of these three plasmids, which needs to be confirmed by further study. Anyway, the cotransfer and coexistence of *mcr-9* and carbapenemase genes in *E. hormaechei* isolates limit the choice of antibiotics, which will arise a huge risk to clinical treatment and global public health. Further surveillance is necessary to achieve better insight into the prevalence and dissemination mechanism of these coexisting *bla*_KPC-2_-, *bla*_NDM-1_-, and *mcr-9*-harboring plasmids among clinical isolates.

## Data availability statement

The datasets presented in this study can be found in online repositories. The names of the repository/repositories and accession number(s) can be found at: Genbank- [CP114571 (chromosome), CP114573 (pE1532-KPC), CP114574 (pE1532-MCR), CP114575 (pE1532-NDM), and CP114572 (pE1532-4)].

## Ethics statement

The bacterial isolate involved in this study was part of the routine hospital laboratory procedure, and the clinical information of the patient was not involved in this study, so ethical approval and informed consent were not required.

## Author contributions

WF and FS conceived and designed the study. LRX and SL carried out the phenotypic characterization. QY and WF carried out the genomic analysis and bioinformatics analysis. WF, QY and FS wrote and revised the manuscript. PX and LLX did the funding acquisition. All authors contributed to the article and approved the submitted version.

## Funding

This work was supported by the Innovation Leading Talent Project of Chongqing (425Z2P12D) and Medical Service Nursery Talent Program of Army Medical University (XZ-2019-505-056).

## Conflict of interest

The authors declare that the research was conducted in the absence of any commercial or financial relationships that could be construed as a potential conflict of interest.

## Publisher’s note

All claims expressed in this article are solely those of the authors and do not necessarily represent those of their affiliated organizations, or those of the publisher, the editors and the reviewers. Any product that may be evaluated in this article, or claim that may be made by its manufacturer, is not guaranteed or endorsed by the publisher.

## Footnotes

^1^ http://pubmlst.org/ecloacae/

^2^ https://inkscape.org/en/

^3^ http://www.eucast.org/
