# Supplementary material for: First report of coexistence of blaKPC-2-, blaNDM-1- and mcr-9-carrying plasmids in a clinical carbapenem-resistant Enterobacter hormaechei isolate
Source: Front Microbiol. 2023 Mar 23;14:1153366. doi: 10.3389/fmicb.2023.1153366 (PMC10076803; doi:10.3389/fmicb.2023.1153366)
Supplement: Supplementary file 1 [file Data_Sheet_1.docx]

***Supplementary Material***

**First report of coexistence of *bla*_KPC-2_-, *bla*_NDM-1_- and *mcr-9*-carrying plasmids in a clinical carbapenem-resistant *Enterobacter hormaechei* isolate**

Qian Yuan, Peiyuan Xia, Lirong Xiong, Linli Xie, Shan Lv, Fengjun Sun* and Wei Feng*

Department of Pharmacy, Southwest Hospital, Third Military Medical University (Army Medical University), Chongqing 400038, China

***Correspondence:**

Fengjun Sun, [fengj_sun@163.com](mailto:fengj_sun@163.com,)

Wei Feng, [fengwei.sky@163.com](mailto:fengwei.sky@163.com)

**Supplementary Figures:**

**Supplementary Figure S1.** Schematic maps of sequenced plasmids pE1532-KPC, pE1532-NDM, pE1532-MCR and pE1532-4. Genes are denoted by arrows and colored based on gene functional classification. The innermost circle presents the GC skew [(G− C)/(G+C)] with a window size of 500 bp and a step size of 20 bp. The next-to-innermost circle presents the GC content.

**Supplementary Figure S2.** Linear comparison of plasmid pE1532-MCR with R478 and p505108-MDR. Shown are linear comparison of the three sequenced plasmids pE1532-MCR, R478 (accession number BX664015) and p505108-MDR (accession number KY978628) (A) and that of the *ΔklaB* to *orf819* regions of these three plasmids (B). Genes are denoted by arrows. Genes, mobile elements and other features are colored based on functional classification. Shading denotes shared regions of homology (>95% nucleotide identity). Numbers in brackets indicate nucleotide positions within the corresponding plasmids.

**Supplementary Figure S3.** Tn*6362* (accession number KY978628) and Tn*2* (accession number HM749967) from pE1532-MCR. Genes are denoted by arrows and colored based on gene functional classification. Numbers in brackets indicate nucleotide positions within the corresponding plasmids.

**Supplementary Table:**

**Supplementary Table S1.** The *bla*_NDM-1_-harboring IncX3 plasmids selected with the highest identity to pE1532-NDM backbone sequences and with the different origins of bacterial species or hosts

| Plasmid | Strain | Host | Location | Similarity | Coverage | GenBank Accession |
| --- | --- | --- | --- | --- | --- | --- |
| pE1532-NDM | *Enterobacter hormaechei* | Human | China | - | - | CP114575 |
| p112298-NDM | *Citrobacter freundii* | Human | China | 100% | 100% | KP987216.1 |
| pEC10-NDM-1 | *Escherichia coli* | Human | China | 100% | 100% | CP060949.1 |
| pMTC948 | *Escherichia coli* | Shrimp | Unknown | 99.94% | 100% | MH349095.1 |
| pHNAH566 | *Escherichia coli* | Chicken | China | 100% | 100% | MH286946.1 |
| pABC40-NDM-1 | *Enterobacter cloacae* | Human | Unknown | 100% | 100% | MK372380.1 |
| pEk72-3 | *Enterobacter kobei* | Human | China | 100% | 100% | CP088232.1 |
| pNDM1_095845 | *Klebsiella pneumoniae* | Human | China | 100% | 100% | CP031884.1 |
| pSL131_IncA/C-IncX3 | *Salmonella enterica* | Human | China | 100% | 100% | MH105050.1 |
| pNDM1_SCW13 | *Kluyvera cryocrescens* | Hospital sewage | China | 100% | 99% | MN178638 |
| pABC140-NDM-1 | *Morganella morganii* | Human | United Arab Emirates | 100% | 100% | MK372385 |
| pRor-30818cz | *Raoultella ornithinolytica* | Unknown | Unknown | 100% | 100% | MG252893 |
