# Supplementary figures and images for: First report of coexistence of blaKPC-2-, blaNDM-1- and mcr-9-carrying plasmids in a clinical carbapenem-resistant Enterobacter hormaechei isolate
Source: Front Microbiol. 2023 Mar 23;14:1153366. doi: 10.3389/fmicb.2023.1153366 (PMC10076803; doi:10.3389/fmicb.2023.1153366)

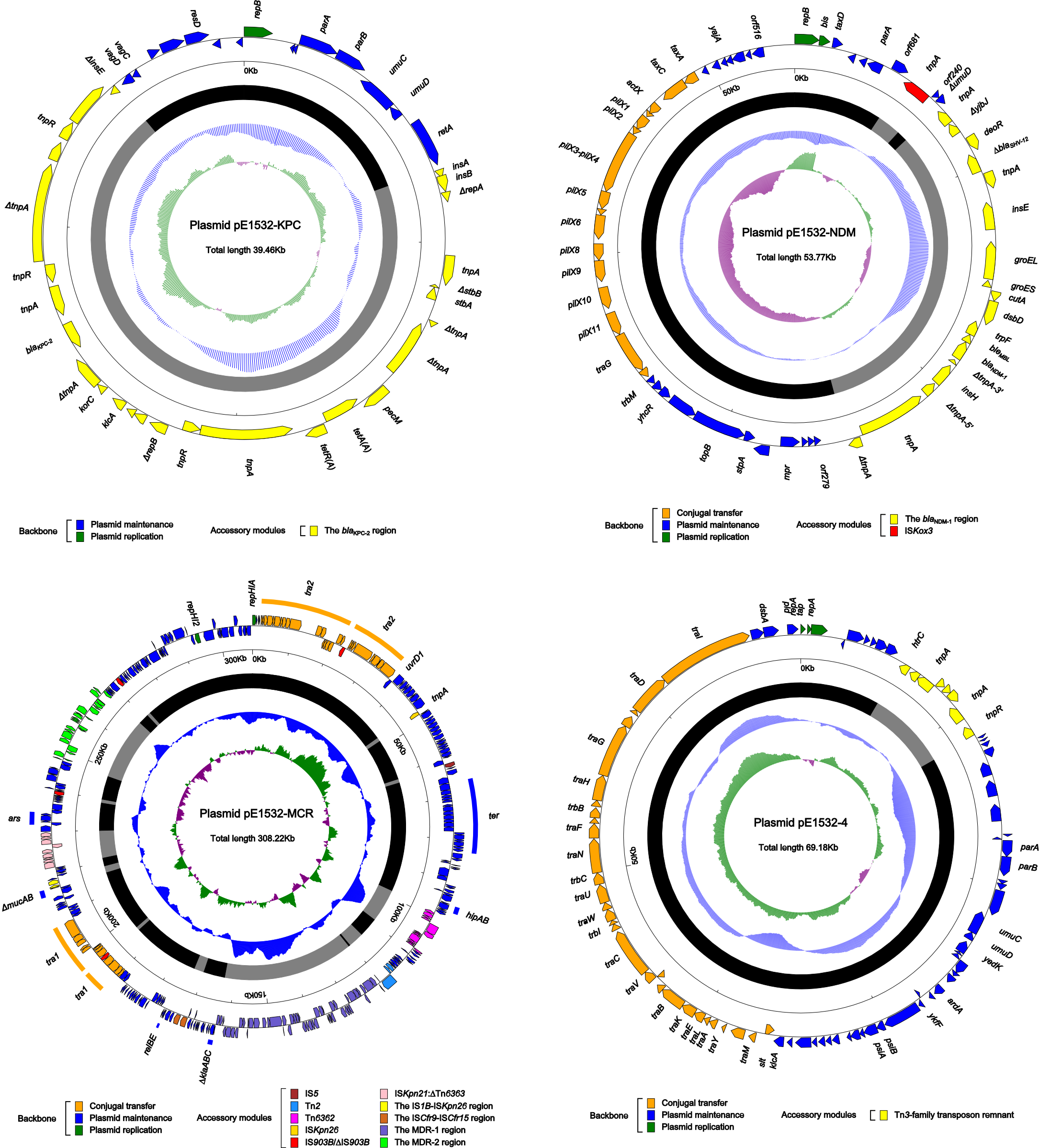

Supplement: Supplementary file 2 [file Image_1.TIF]

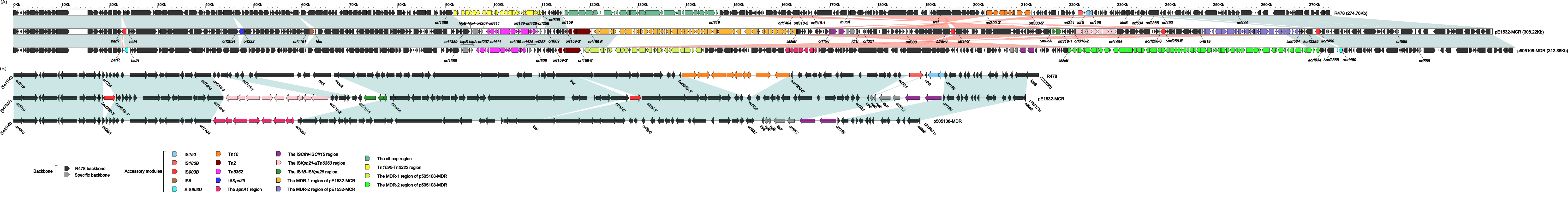

Supplement: Supplementary file 3 [file Image_2.TIF]

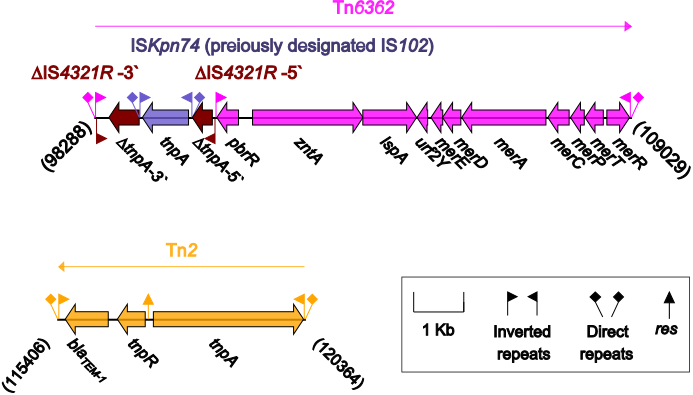

Supplement: Supplementary file 4 [file Image_3.TIF]
